# Association between *Helicobacter pylori* infection and MASLD: a cross-sectional study with interpretable machine learning for MASLD risk prediction

**DOI:** 10.3389/fnut.2026.1730461

**Published:** 2026-06-22

**Authors:** Yue Zhang, Ruifeng Duan, Yuzhong Zhang, Lijuan Wei

**Affiliations:** Department of Gastroenterology and Digestive Endoscopy Center, The Second Hospital of Jilin University, Changchun, China

**Keywords:** *Helicobacter pylori*, inflammatory index, machine learning, steatotic liver disease, triglyceride glucose

## Abstract

**Background:**

*Helicobacter pylori* (HP) infection has been increasingly linked to metabolic dysfunction-associated steatotic liver disease (MASLD). However, evidence remains inconsistent, and the predictive value of HP status within integrated risk models is unclear. This study aimed to evaluate the association between HP infection and MASLD and to develop interpretable machine learning models for MASLD risk stratification.

**Methods:**

A total of 1,021 participants from a Chinese hospital-based cohort and 4,870 participants from the U.S. National Health and Nutrition Examination Survey (NHANES 1999–2000) were included. In the Chinese cohort, active HP infection was assessed using the 13C or 14C-urea breath test, and MASLD was diagnosed by ultrasonography. In NHANES, HP exposure was defined by serum IgG seropositivity, and MASLD was approximated using the Fatty Liver Index (FLI). Logistic regression was used to assess the association between HP and MASLD. Mediation analysis was conducted to explore potential metabolic and inflammatory pathways. Eleven machine learning models were developed in the Chinese cohort and evaluated using ROC, calibration, and SHAP analysis, with external comparison in NHANES using a reduced feature set to minimize potential bias.

**Results:**

In the Chinese cohort, HP infection was significantly associated with MASLD in crude (OR = 7.11), age- and sex-adjusted (OR = 6.48), and further adjusted models (including diabetes, cholesterolemia, and BMI; OR = 4.83, 95% CI: 3.07–7.23, *p* < 0.001). This elevated OR should be interpreted as cohort-specific and not generalizable. A positive association was also observed in NHANES (adjusted OR = 1.22, 95% CI: 1.14–1.59, *p* = 0.017). Machine learning models demonstrated strong internal performance (AUC up to 0.8876), while external comparison in NHANES showed moderate discrimination (best AUC = 0.6536). SHAP analysis identified HP and LHR as the top contributors to MASLD prediction, with TyG-related indices showing secondary importance. Mediation analysis suggested potential involvement of metabolic and inflammatory indices, although these findings should be interpreted as exploratory.

**Conclusion:**

HP infection was associated with MASLD in both Chinese and U.S. populations. Interpretable machine learning models demonstrated potential for MASLD risk stratification, with consistent identification of key predictors across cohorts. However, due to cross-sectional design, differences in diagnostic definitions, and the use of FLI in NHANES, findings should be interpreted as associative rather than causal, and external validation results should be considered exploratory.

## Introduction

*Helicobacter pylori* (HP) is a common pathogenic microorganism in the gastrointestinal tract, primarily colonizing the gastric antrum, and has been classified as a Group I carcinogen (1). Epidemiological studies indicate that more than 50% of the global population is infected with HP (2, 3). Emerging evidence suggests that HP infection may also be associated with a spectrum of metabolic disorders, including obesity, impaired glucose metabolism, dyslipidemia, and metabolic dysfunction–associated steatosis liver disease (MASLD) (4, 5).

MASLD, previously referred to as nonalcoholic fatty liver disease (NAFLD), is characterized by hepatic fat accumulation exceeding 5% of liver content and has been recognized as a major global public health concern (6). Its pathogenesis involves a complex interplay of metabolic factors, progressing from simple steatosis to non-alcoholic steatohepatitis (NASH) and potentially to fibrosis and cirrhosis (7). The pathogenesis of MASLD involves insulin resistance and genetic predispositions that profoundly disrupt normal physiological processes and may adversely affect patients’ quality of life (8, 9). For instance, the liver-derived glycoprotein fetuin-A, which impairs insulin signaling, has been found to be elevated in individuals with NAFLD, potentially linking hepatic steatosis with increased cardiometabolic risk (10). Recent studies have highlighted that HP infection may be associated with the onset and progression of MASLD through mechanisms such as increased oxidative stress, systemic inflammation, and altered lipid metabolism (11).

Despite these advances, significant gaps remain in the literature regarding the relationship between HP infection and metabolic dysregulation (12). The possible association between HP infection and NAFLD has been proposed at genetic, molecular, and clinical levels, yet findings remain heterogeneous (13). First, a recent meta-analysis demonstrated a positive association between HP infection and MASLD (2). Similarly, studies by Tang et al. (13) and Polyzos et al. (14) identified HP infection as an independent risk factor for MASLD progression. However, subgroup analyses revealed no significant difference in HP infection rates between patients with simple steatosis and those with advanced MASLD, suggesting that HP infection alone may not be a direct or sufficient driver of disease progression. Second, most existing studies have focused on isolated metabolic parameters—such as body mass index (BMI) or blood glucose—rather than a comprehensive evaluation of multiple metabolic factors within the same population (15). Moreover, epidemiological evidence remains inconsistent, and the biological mechanisms underlying the HP and MASLD association are still poorly understood. Few studies have examined how metabolic and inflammatory indices jointly mediate this relationship or whether machine learning approaches can effectively predict the MASLD risk.

Therefore, the present study aimed to (1) investigate the association between HP infection and MASLD in both a Chinese cohort and the U.S. NHANES population; (2) identify key metabolic and inflammatory factors associated with MASLD Risk Prediction; and (3) develop and compare multiple machine learning models to predict the MASLD risk. Additionally, Shapley Additive explanations (SHAP) analysis was employed to determine the most influential predictors and enhance model interpretability. This study provides novel insights into the metabolic and inflammatory mechanisms linking HP infection and MASLD and offers a data-driven framework for early risk identification and precision prevention.

## Method

### Study population

**Figure 1:**
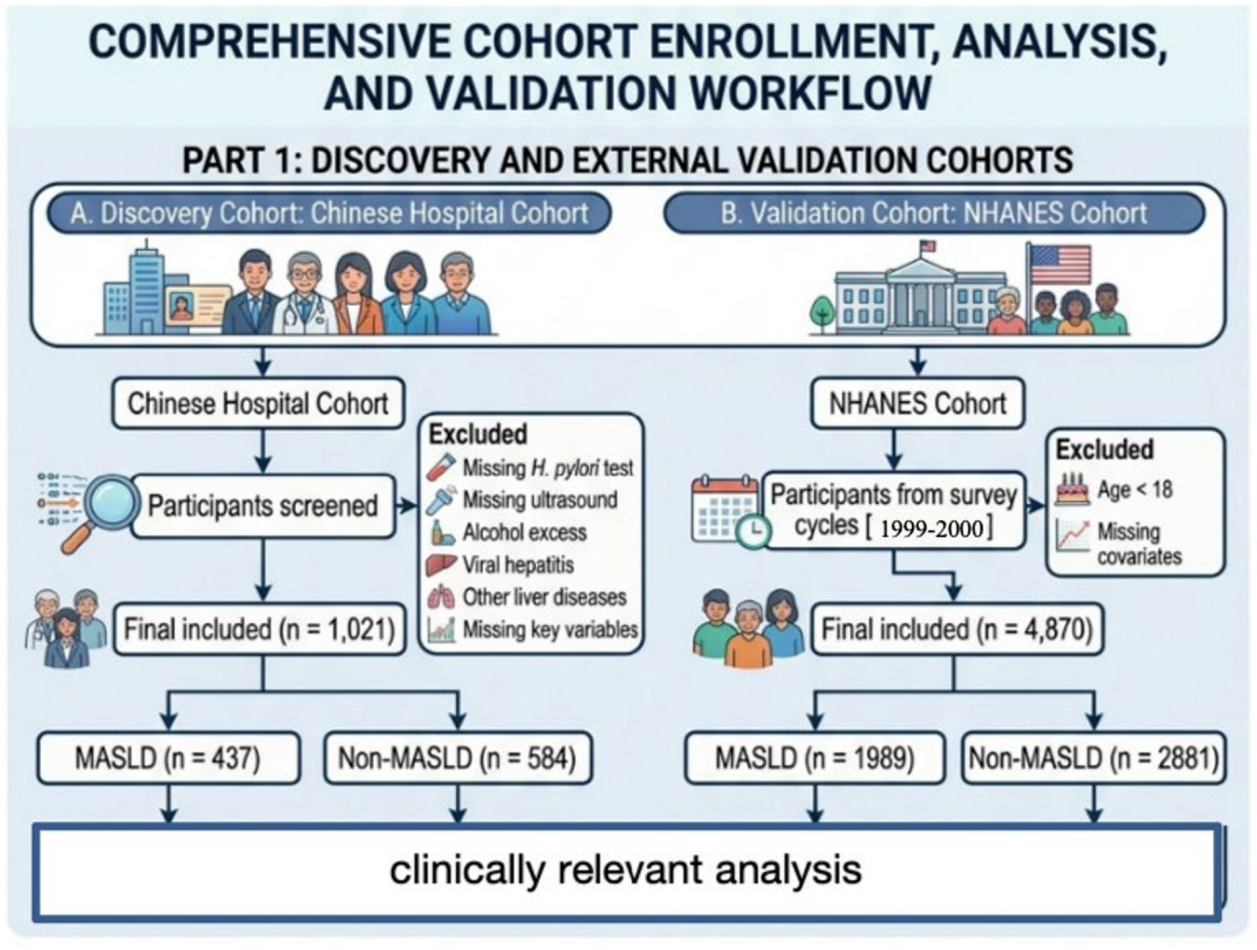
Study flowchart of participant selection, cohort construction, and analytical workflow.

Chinese participants were recruited from individuals who underwent both the 14C or 13C-urea breath test (UBT) and abdominal ultrasonography at the Health Examination Center of the Second Hospital of Jilin University between January 2024 and May 2025. Participants with complete demographic and clinical data were included, including sex, age, fasting plasma glucose, lipid profile [total cholesterol, triglycerides (TG), low-density lipoprotein cholesterol (LDL-C), high-density lipoprotein cholesterol (HDL-C)], liver function, and complete blood counts (Figure 1). The exclusion criteria were as follows: age < 18 years; hepatic steatosis due to non-metabolic causes (e.g., viral hepatitis, alcohol consumption, or drug-induced liver injury); heavy alcohol intake within the past 2 years (men > 21 drinks/week, women > 14 drinks/week); history of cancer, gastrectomy, or severe hepatic/renal dysfunction. The study protocol was approved by the Ethics Committee of the Second Hospital of Jilin University.

For external comparison, data from the U.S. National Health and Nutrition Examination Survey (NHANES 1999–2000) were included, the survey cycle in which *Helicobacter pylori* serological data were available. Similar exclusion criteria were applied, including excessive alcohol consumption, viral hepatitis (hepatitis B surface antigen or hepatitis C antibody positivity), and missing key variables. A total of 4,870 participants were included in the final analysis. The NHANES protocol was approved by the Institutional Review Board of the Centers for Disease Control and Prevention (CDC). This study was conducted in accordance with the Declaration of Helsinki and Good Clinical Practice (GCP) guidelines (Figure 1).

### Detection of HP infection

In the Chinese cohort, participants fasted for at least 4 hours prior to testing and avoided antibiotics or proton pump inhibitors for at least 1 month. HP infection was assessed using the 14C or 13C-urea breath test (UBT), with positive results indicating active infection according to established diagnostic criteria (16).

In the NHANES cohort, HP exposure was assessed using serum IgG enzyme-linked immunosorbent assay (ELISA). Participants were classified as HP seropositive (optical density ≥ 1.1) or seronegative (optical density < 0.9), with equivocal results (0.9–1.1) excluded (17, 18).

Importantly, these methods reflect different biological states (active infection vs. prior exposure), which may introduce heterogeneity when comparing results across cohorts.

### Assessment of MASLD

In the Chinese cohort, participants fasted for 10–12 h prior to abdominal ultrasonography. Liver steatosis was assessed by experienced sonographers using a standardized protocol (15, 17).

In the NHANES cohort, hepatic steatosis was approximated using the Fatty Liver Index (FLI), a validated non-invasive surrogate combining anthropometric and biochemical parameters (19). The formula is:

$$\mathrm{FLI}=\frac{e^{\left(\begin{array}{l} 0.953\times \ln\;(\mathrm{TG})+0.139\times \mathrm{BMI}+0.718\\ \times \ln(\mathrm{GGT})+0.053\times \mathrm{WC}-15.4751 \end{array}\right)}}{\left(1+e^{\left(\begin{array}{l} 0.953\times \ln\;(\mathrm{TG})+0.139\times \mathrm{BMI}+0.718\\ \times \ln(\mathrm{GGT})+0.053\times \mathrm{WC}-15.4751 \end{array}\right)}\right)}\times 100$$

FLI ≥ 60 was used to indicate a high probability of hepatic steatosis (20). In the NHANES 1999–2000 cycle, imaging-based assessment of hepatic steatosis (ultrasound or CT) was not available. Therefore, we used the Fatty Liver Index (FLI), a validated non-invasive surrogate that has been widely employed in large epidemiologic studies to estimate the probability of hepatic steatosis (20). FLI is a validated surrogate, but it is not equivalent to ultrasound and does not directly visualise hepatic fat. It relies on indirect markers (TG, BMI, GGT, WC) that can be influenced by other metabolic conditions.

Because FLI incorporates TG and waist circumference, variables derived from these components may introduce information leakage when used as predictors. Therefore, in external validation analyses, features directly related to TG and WC were excluded to reduce potential bias. Specifically, we excluded TG, WC, BMI (when derived from FLI components), and any variable that is a direct component of the FLI formula. Although TyG and TyG/HDL are not direct components of the FLI formula, they are mathematically derived from TG and glucose, which are part of FLI. Therefore, some degree of metabolic overlap is unavoidable, and the external validation results should be interpreted with this limitation in mind.

### Assessment of covariates

Demographic variables (age, sex), anthropometric measures (BMI), and metabolic indicators (fasting glucose, TG, HDL-C, LDL-C) were collected. Composite metabolic and inflammatory indices were calculated as follows (21):

TyGindex=ln[TG×glucose/2]

TyG/HDL−C=TyG/HDL−C

SIRI=(neutrophils×monocytes)/lymphocytes

WNR=white blood cells/neutrophils

WLR=white blood cells/lymphocytes

NLR=neutrophils/lymphocytes

WMR=white blood cells/monocytes

NMR=neutrophils/monocytes

WBC/HDL−C=white blood cells/HDL−C

LHR=lymphocytes/HDL−C

NHR=neutrophils/HDL−C

LSR=AST/ALT

### Statistical analysis

Continuous variables were expressed as median (interquartile range, IQR) and compared using the Mann–Whitney U test. Categorical variables were presented as number (percentage) and compared using the chi-square test. In the cohort, LHR had 34 missing values (0.7%) (Supplementary Table S5). Multiple imputation by chained equations (MICE) was performed using the Iterative Imputer in Python (scikit-learn v1.3). Five imputations were generated with 10 iterations each, using all other complete variables (Age, Sex, HP, DM, Cholesterolemia, TyG, and MASLD) as predictors. Rubin’s rules were applied to combine results. Complete-case sensitivity analysis confirmed the robustness of findings. The association between HP infection and MASLD was evaluated using logistic regression models, reported as odds ratios (ORs) with 95% confidence intervals (CIs). To avoid overadjustment, primary models focused on confounders (e.g., age, sex), while extended models including additional clinical variables were interpreted as exploratory.

Model 1: unadjusted.

Model 2 (China): adjusted for age and sex.

Model 2 (NHANES): adjusted for age, sex, education, marital status.

Model 3 (China): additionally adjusted for clinical variables (exploratory).

Model 3 (NHANES): not used to avoid overadjustment and potential bias.

### Mediation analysis

To explore potential pathways linking HP infection and MASLD, mediation analyses were conducted using selected metabolic and inflammatory indices, including SIRI, WHR, MHR, NHR, LHR, WMR, NMR, LSR, TyG, and TyG/HDL. These mediators were selected based on prior literature linking HP infection to systemic inflammation and insulin resistance, as well as on univariable associations with both HP and MASLD in the current dataset. Analyses were performed in R using the mediation package. A Bayesian approach with 1,000 bootstrap simulations was employed to estimate indirect effects and their 95% confidence intervals, providing robust uncertainty estimation.

All indirect effects were standardized to facilitate comparison across mediators with differing units. Models adjusted for age and sex to control for potential confounding. The estimated indirect effects *β* represent the contribution of each mediator to the association between HP and MASLD, whereas the direct effect reflects the residual association not explained by the mediator.

Mediation analysis assumes sequential ignorability (i.e., no unmeasured confounding of the exposure-mediator and mediator-outcome relationships). These assumptions cannot be fully verified in cross-sectional data. Given the cross-sectional design, mediation results should be interpreted as exploratory and hypothesis-generating, not causal. The approach provides preliminary insight into potential metabolic and inflammatory pathways but cannot establish temporality or mechanistic causation. Visual representation of the mediation results was provided as a forest plot, with each mediator’s point estimate and 95% confidence interval indicated.

### Machine learning

This study utilized a hospital-based Chinese cohort to predict MASLD risk. Five key features were included in modeling and SHAP analysis: HP infection status, LHR, age, sex, and diabetes mellitus (DM). Feature selection was performed *a priori* based on clinical relevance and prior literature, not using any data-driven method (e.g., stepwise selection or recursive feature elimination) on the training set. Therefore, no feature selection-induced overfitting occurred. Categorical variables were appropriately encoded, and continuous variables were converted to numeric type. Variance inflation factor (VIF) was calculated for the five selected features; all VIF values were <2, indicating no severe multicollinearity. Missing values were imputed within the training set only (median for numeric variables, mode for categorical variables) to avoid information leakage, and the same transformations were applied to the test set using parameters learned from the training set.

The cohort was split into 70% training and 30% testing sets. Within the training set, 5-fold cross-validation was employed for hyperparameter assessment. For GBM, hyperparameters were assessed using 5-fold CV on the training set (Supplementary Table S1). Due to computational constraints, we did not perform an exhaustive grid search for all models. Instead, we used default or fixed hyperparameters (Supplementary Table S1) for most algorithms and evaluated their performance using 5-fold CV. For GBM, a limited assessment was performed (Supplementary Table S1). Within the 5-fold cross-validation used for hyperparameter assessment, the same preprocessing (imputation and scaling) was re-fitted on each training fold and applied to the corresponding validation fold. This ensures no information from the validation or test sets leaks into the model training. Final models were retrained on the full training set. Comparisons of AUCs between models were conducted using DeLong’s test with bootstrap resampling (Supplementary Table S4).

**Figure 2:**
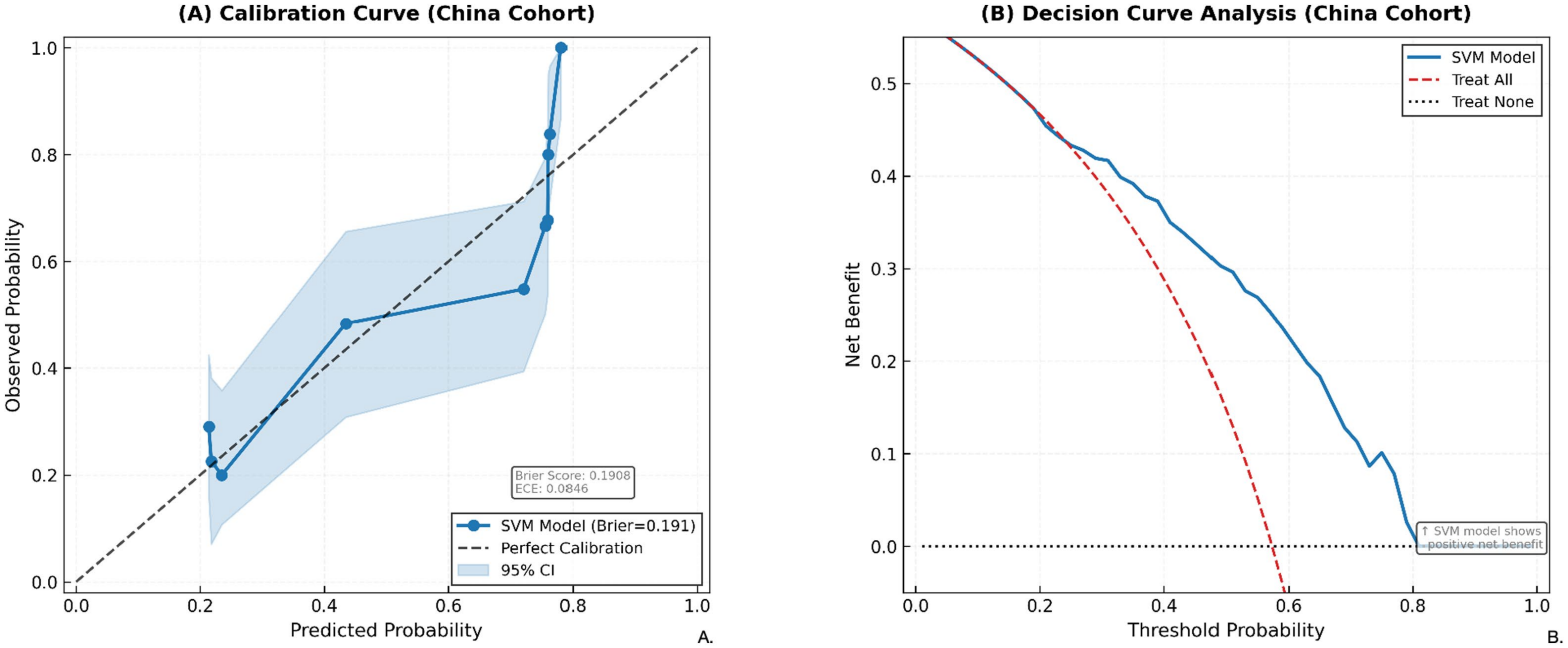
Calibration and clinical utility of the machine learning prediction model. **(A)** Calibration plot showing the agreement between predicted probabilities and observed outcomes. The diagonal 45-degree line represents perfect calibration. The blue points with error bars indicate the mean predicted risk and corresponding 95% confidence intervals across deciles. **(B)** Decision curve analysis (DCA) illustrating the net clinical benefit of the prediction model (“pred”) across a range of threshold probabilities, compared with the “treat-all” and “treat-none” strategies.

Model performance was evaluated on the independent test set using AUC, accuracy, F1-score, precision, and recall. Calibration was assessed using the calibration curve (Figure 2A), which plots the predicted probabilities against the observed outcomes; the curve’s proximity to the 45-degree diagonal line indicates good calibration. Decision curve analysis (DCA) demonstrated that the model achieved higher net benefit across threshold probabilities compared with “treat-all” or “treat-none” strategies. SHAP analysis interpreted feature importance, showing LHR as the most influential predictor, followed by HP infection, age, sex, and DM. SHAP values represent predictive contribution, not causality.

External validation was conducted using the NHANES cohort. Differences in MASLD definitions (ultrasound in the Chinese cohort vs. Fatty Liver Index in NHANES) and HP assessment (active infection vs. serology) were recognized as limitations potentially affecting model generalizability. LHR missing data (0.7%) in NHANES was handled via multiple imputation by chained equations (MICE) with 5 imputations and up to 10 iterations per imputation. Sensitivity analyses using complete cases yielded consistent results (Supplementary Table S5).

Analyses were discussed with a biostatistician/ML expert to ensure methodological rigor. “For software versions, we now specify: Python 3.10, scikit-learn 1.3”, XGBoost 1.7, SHAP 0.42, matplotlib 3.7. Analyses were discussed with a biostatistician/ML expert to ensure methodological rigor.

## Results

A total of 1,021 participants were included. Table 1 presents baseline characteristics stratified by MASLD status. The HP-positive group comprised 584 participants (57.2%), and the HP-negative group 427 (41.8%). The median age of participants in non-HP group was 38.00 years (Q₁: 32.00, Q₃: 50.00), while in HP group, it was 43.00 years (Q₁: 35.00, Q₃: 52.00), showing a significant age difference between the two groups (Z = −3.95, *p* < 0.001). Additionally, a total of 4,870 participants from the NHANES dataset were included in this study, with 1989 individuals in the “Yes” group (HP infected) and 2,881 individuals in the “No” group (non-HP infected) (Supplementary Table S2). In the NHANES dataset, the “Yes” group (HP infected) was significantly older than the “No” group. A higher proportion of females were found in the “Yes” group, and there were notable differences in education levels and marital status. The “Yes” group had a higher proportion of individuals with lower education levels and a higher percentage of married individuals compared to the “No” group. The “Yes” group showed a higher prevalence of obesity, diabetes, and elevated triglycerides compared to the “No” group. Several metabolic and inflammatory indices were significantly higher in the “Yes” group, including the TyG index, TyG/HDL ratio, SIRI, and NLR. The “Yes” group also exhibited higher values for various ratios such as WBC/HDL-C, MHR, NHR, and LHR, which are indicative of increased metabolic dysfunction and systemic inflammation.

**Table 1:** Baseline characteristics of the Chinese cohort stratified by MASLD status.

Variables	Total (*n* = 1,021)	No (*n* = 437)	Yes (*n* = 584)	Statistic	*p*
Age, M (Q1, Q3)	42.00 (34.00, 52.00)	38.00 (32.00, 50.00)	43.00 (35.00, 52.00)	*χ*^2^ = 2.01	0.156
Gender, *n* (%)				*χ*^2^ = 70.12	<0.001
Woman	574 (56.22)	180 (41.19)	394 (67.47)		
Man	447 (43.78)	257 (58.81)	190 (32.53)		
HP, *n* (%)				*χ*^2^ = 207.14	<0.001
No	427 (41.82)	295 (67.51)	132 (22.60)		
Yes	594 (58.18)	142 (32.49)	452 (77.40)		
Diabetes, *n* (%)				*χ*^2^ = 13.58	<0.001
No	977 (95.69)	430 (98.40)	547 (93.66)		
Yes	44 (4.31)	7 (1.60)	37 (6.34)		
Cholesterolemia, *n* (%)				*χ*^2^ = 7.96	0.005
No	909 (89.03)	403 (92.22)	506 (86.64)		
Yes	112 (10.97)	34 (7.78)	78 (13.36)		
BMI, M (Q1, Q3)	24.21 (21.48, 26.67)	23.74 (21.45, 26.26)	24.44 (21.72, 27.11)	Z = −2.14	0.032
SIRI, M (Q1, Q3)	0.53 (0.45, 0.75)	0.50 (0.42, 0.68)	0.59 (0.47, 0.79)	Z = −4.66	<0.001
WLR, M (Q1, Q3)	3.00 (2.64, 3.30)	3.00 (2.65, 3.22)	3.00 (2.64, 3.33)	Z = −0.06	0.954
NLR, M (Q1, Q3)	1.68 (1.40, 2.02)	1.68 (1.43, 1.96)	1.68 (1.39, 2.04)	Z = −0.27	0.784
WNR, M (Q1, Q3)	1.79 (1.63, 1.90)	1.79 (1.66, 1.86)	1.79 (1.62, 1.92)	Z = −0.51	0.612
WHR, M (Q1, Q3)	4.24 (3.37, 5.32)	4.09 (3.03, 4.32)	4.53 (3.72, 5.70)	Z = −9.20	<0.001
MHR, M (Q1, Q3)	0.22 (0.19, 0.32)	0.21 (0.17, 0.25)	0.26 (0.21, 0.36)	Z = −9.76	<0.001
NHR, M (Q1, Q3)	2.37 (1.86, 3.06)	2.35 (1.64, 2.50)	2.57 (2.05, 3.33)	Z = −8.14	<0.001
LHR, M (Q1, Q3)	1.41 (1.13, 1.81)	1.37 (1.03, 1.43)	1.53 (1.26, 2.00)	Z = −9.02	<0.001
WMR, M (Q1, Q3)	18.00 (15.00, 20.00)	19.00 (15.33, 20.00)	17.50 (14.67, 20.00)	Z = −2.65	0.008
NMR, M (Q1, Q3)	10.33 (8.22, 11.47)	10.93 (8.53, 11.35)	9.94 (8.06, 11.60)	Z = −2.30	0.021
LSR, M (Q1, Q3)	1.05 (0.82, 1.22)	1.00 (0.73, 1.05)	1.08 (0.90, 1.38)	Z = −9.03	<0.001
TyG, M (Q1, Q3)	3.68 (3.35, 4.04)	3.65 (3.18, 3.68)	3.81 (3.52, 4.26)	Z = −10.45	<0.001
TyG/HDL-C, M (Q1, Q3)	2.60 (2.07, 3.18)	2.49 (1.83, 2.60)	2.81 (2.33, 3.61)	Z = −10.23	<0.001
TyG/LDL-C, M (Q1, Q3)	1.29 (1.12, 1.45)	1.29 (1.16, 1.40)	1.29 (1.10, 1.51)	Z = −0.24	0.807

Z: Mann–Whitney test, *χ*^2^: Chi-square test.

M: Median, Q1: 1st Quartile, Q3: 3rd Quartile.

### The association between HP infection and MASLD

In the Chinese cohort, HP infection was significantly associated with MASLD across all models (Table 2). In the crude model (Model 1), HP-positive individuals had markedly higher odds of MASLD compared with HP-negative individuals (OR = 7.11, 95% CI: 5.38–9.40, *p* < 0.001). After adjustment for age and sex (Model 2), the association remained robust (OR = 6.48, 95% CI: 4.86–8.65, *p* < 0.001). In the further adjusted model (Model 3), which additionally included clinical variables such as diabetes, and cholesterolemia, the association between HP infection and MASLD remained statistically significant (OR = 4.83, 95% CI: 3.07–7.23, p < 0.001). Given that some of these additional variables may be related to metabolic status and could lie on the causal pathway, Model 3 should be interpreted as exploratory. Given the potential selection bias and residual confounding, the OR observed in the Chinese cohort (especially the fully adjusted OR of 4.83) should be interpreted as cohort-specific and not directly extrapolated to other populations or to the general population.

**Table 2:** Multivariable logistic regression analysis for MASLD risk (*β*, 95% CI) (China).

Variables	Model 1		Model 2		Model 3	
	OR (95% CI)	*p*	OR (95% CI)	*p*	OR (95% CI)	*p*
HP						
No	1.00 (Reference)		1.00 (Reference)		1.00 (Reference)	
Yes	7.11 (5.38 ~ 9.40)	<0.001	6.48 (4.86 ~ 8.65)	<0.001	4.83 (3.07 ~ 7.23)	<0.001

OR, odds ratio; CI, confidence interval.

Model 1: crude.

Model 2: adjust: age, gender.

Model 3 adjusted for age, gender, diabetes, cholesterolemia, and BMI.

In the NHANES cohort, a positive association between HP seropositivity and MASLD was also observed (Table 3), although the effect size was substantially smaller compared with the Chinese cohort. In the crude model (Model 1), HP seropositivity was associated with an increased risk of MASLD (OR = 1.09, 95% CI: 1.01–1.53, *p* = 0.040). After adjustment for age, sex, education, and marital status (Model 2), the association remained statistically significant (OR = 1.22, 95% CI: 1.14–1.59, *p* = 0.017). However, given the differences in HP assessment (active infection vs. seropositivity) and MASLD definition (ultrasound vs. FLI), these findings should be interpreted cautiously as cross-sectional associative evidence rather than direct replication.

**Table 3:** Multivariable logistic regression analysis for MASLD risk (*β*, 95% CI) (NHANES).

Variables	Model 1		Model 2	
	OR (95% CI)	*p*	OR (95% CI)	*p*
HP				
No	1.00 (Reference)		1.00 (Reference)	
Yes	1.09 (1.01 ~ 1.53)	0.04	1.22 (1.14 ~ 1.59)	0.017

OR, odds ratio, CI, confidence interval.

Model 1: crude.

Model 2: adjust: age, gender, education, marital.

Overall, HP infection showed a consistent positive association with MASLD across two independent cohorts, although the magnitude of association varied and should be interpreted in the context of differing diagnostic approaches.

### Mediation analysis

**Figure 3:**
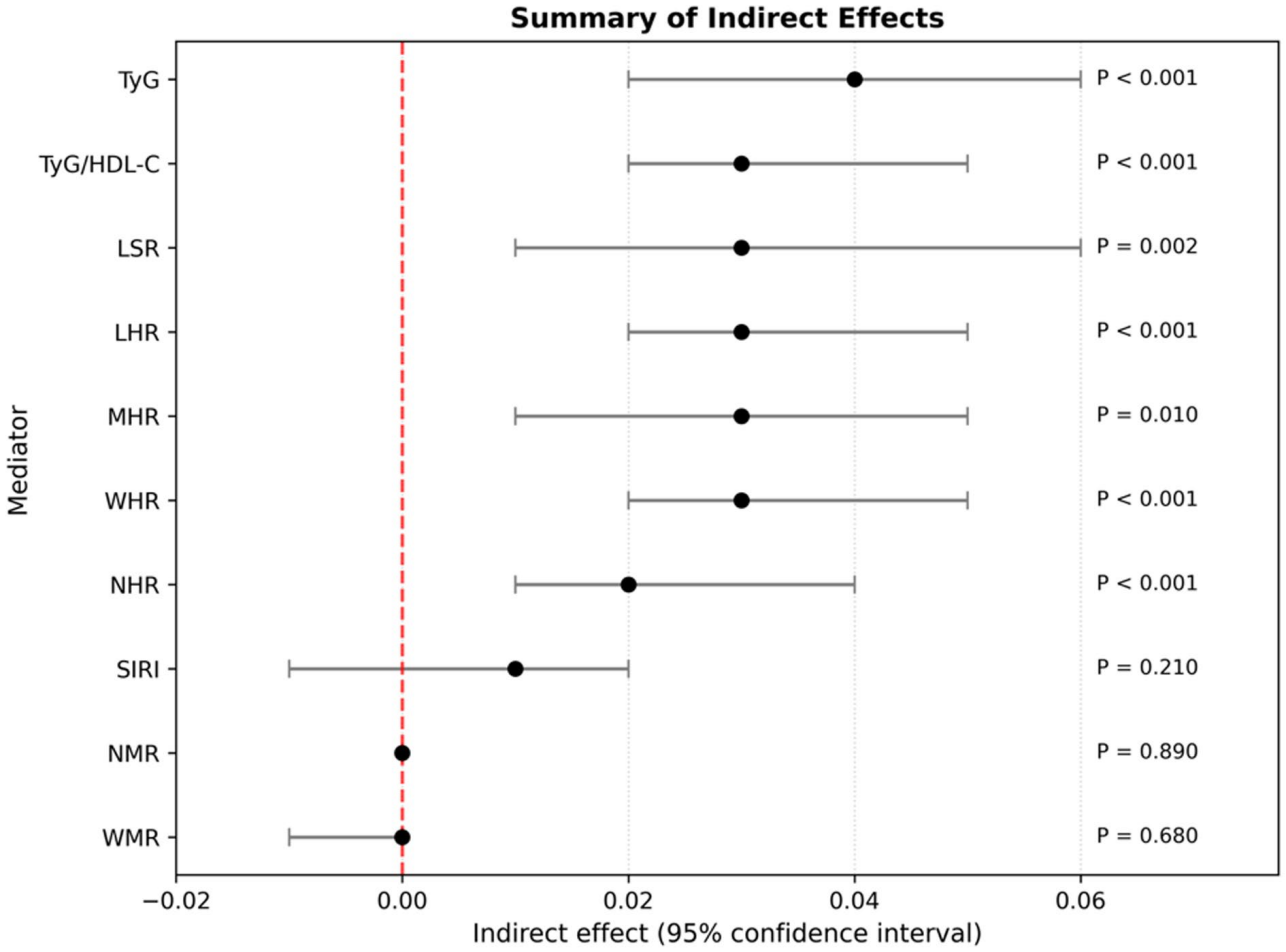
Mediation analysis of the association between HP infection and MASLD through metabolic and inflammatory indices.

To explore potential pathways linking HP infection and MASLD, mediation analyses were performed using selected metabolic and inflammatory indices (Figure 3). Several indices, including TyG, TyG/HDL-C, LHR, NHR, and MHR, showed significant indirect effects, suggesting that metabolic dysregulation and systemic inflammation may partially account for the observed association between HP infection and MASLD. In contrast, some markers such as SIRI did not demonstrate statistically significant mediation effects. Given the cross-sectional nature of the data, these findings should be interpreted as exploratory and hypothesis-generating rather than evidence of causal mediation.

Subgroup analyses were conducted to assess whether the association between HP infection and MASLD differed across key clinical characteristics (Supplementary Table S3). The association between HP infection and MASLD was consistent across diabetes subgroups, with no significant interaction (P for interaction = 0.502). HP infection was significantly associated with MASLD among participants without diabetes (OR = 7.07, 95% CI: 5.32–9.39, *p* < 0.001), whereas the association was not statistically significant among those with diabetes (OR = 3.87, 95% CI: 0.68–21.93, *p* = 0.126), likely due to the small sample size. No significant interaction was observed across age groups (P for interaction = 0.348). HP infection was associated with MASLD both in participants aged ≤42 years (OR = 7.79, 95% CI: 5.25–11.56, *p* < 0.001) and >42 years (OR = 5.95, 95% CI: 3.99–8.88, *p* < 0.001), indicating a consistent association across age strata. A significant interaction by sex was observed (P for interaction = 0.002). The association between HP infection and MASLD was stronger in men (OR = 10.85, 95% CI: 6.95–16.94, *p* < 0.001) compared with women (OR = 4.32, 95% CI: 2.96–6.29, *p* < 0.001).

### Machine learning

**Figure 4:**
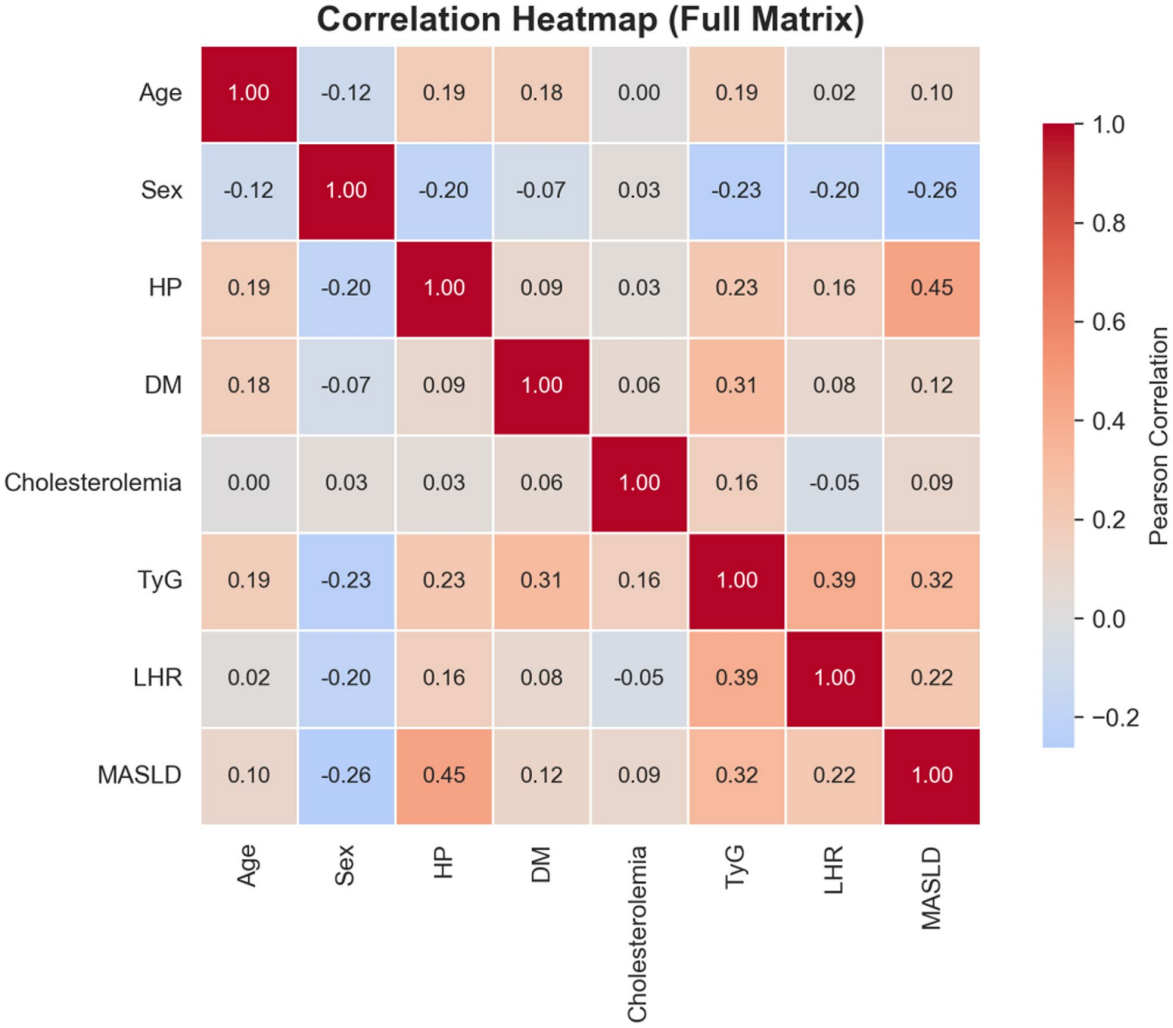
Correlation heatmap of clinical, metabolic, and inflammatory variables in the Chinese cohort. The heatmap displays Pearson correlation coefficients among key variables, including age, sex, HP infection, DM, cholesterolemia, TyG index, LHR, and MASLD status. Color intensity represents the strength and direction of correlations (red: positive; blue: negative).

Correlation analysis was performed to assess relationships among clinical, metabolic, and inflammatory variables in the Chinese cohort (Figure 4). HP infection showed a moderate positive correlation with MASLD, while metabolic indices such as TyG and inflammatory markers such as LHR also demonstrated positive associations with MASLD. Overall, correlations among predictor variables were weak to moderate, suggesting no severe multicollinearity among the selected features used in the simplified prediction models.

The performance of multiple machine learning models for predicting MASLD in the Chinese cohort is summarized in Table 4. Among all models, the GBM achieved the best performance, with an AUC of 0.8876, followed closely by Random Forest (RF, AUC = 0.8803) and C5.0 (AUC = 0.8795). In external validation using the NHANES cohort, model performance decreased across all algorithms compared with the Chinese cohort. The NB model achieved the highest AUC (0.6536), followed by MLP (AUC = 0.6008) and logistic regression (AUC = 0.5887). Overall, discrimination ability in the NHANES cohort was modest, with AUC values ranging from 0.5454 to 0.6536. This reduction in performance may reflect differences in population characteristics, diagnostic methods (ultrasound and FLI), and HP assessment (active infection and seropositivity), as well as the use of a simplified feature set to avoid potential data leakage.

**Table 4:** Model performance comparison for predicting MASLD risk.

Model	AUC	Recall	Precision	Accuracy	Specificity	F1	Kappa
China cohort							
GBM	0.8876	0.8466	0.8514	0.8274	0.8015	0.849	0.6475
RF	0.8803	0.8239	0.8286	0.8013	0.771	0.8262	0.5943
C5.0	0.8795	0.8523	0.8523	0.8306	0.8015	0.8523	0.6538
XGB	0.8636	0.8011	0.8198	0.785	0.7634	0.8103	0.5623
NN	0.8486	0.7784	0.8405	0.7883	0.8015	0.8083	0.5727
MLP	0.8394	0.8011	0.7966	0.7687	0.7252	0.7989	0.5268
KNN	0.7989	0.7386	0.7647	0.7199	0.6947	0.7514	0.4308
LR	0.7963	0.7784	0.7446	0.7199	0.6412	0.7611	0.4229
GP	0.7961	0.8068	0.7474	0.7329	0.6336	0.776	0.4465
SVM	0.7927	0.8125	0.7333	0.7231	0.6031	0.7709	0.4234
NB	0.7838	0.5909	0.8	0.6808	0.8015	0.6797	0.3756
Nhanes cohort							
NB	0.6536	0.4636	0.5478	0.6241	0.7351	0.5022	0.2035
MLP	0.6008	0.4879	0.5291	0.6129	0.6994	0.5076	0.1896
LR	0.5887	0.5854	0.4656	0.5556	0.535	0.5187	0.1158
XGB	0.5836	0.5455	0.4578	0.5498	0.5528	0.4978	0.0955
KNN	0.5826	0.4692	0.4608	0.5583	0.62	0.4649	0.0889
SVM	0.5674	0.5708	0.4993	0.5904	0.6039	0.5327	0.1709
C5.0	0.5666	0.5435	0.4252	0.5128	0.4916	0.4771	0.0336
RF	0.5647	0.5677	0.4556	0.5457	0.5304	0.5055	0.0946
GP	0.5625	0.4454	0.504	0.5939	0.6966	0.4729	0.1446
NN	0.5581	0.4914	0.4767	0.5713	0.6267	0.4839	0.1175
GBM	0.5454	0.5546	0.4315	0.519	0.4944	0.4854	0.0469

To further evaluate the robustness of selected predictors, we compared model performance using two feature sets: LHR-based and LSR-based models (Supplementary Figure S1). Across all machine learning algorithms, the LHR-based models consistently outperformed LSR-based models in external validation. The best-performing LHR model (NB) achieved an AUC of 0.6536, whereas the best LSR-based model (SVM) achieved a lower AUC of 0.5264. These findings suggest that LHR may provide more stable and generalizable predictive information than LSR in cross-cohort validation.

**Figure 5:**
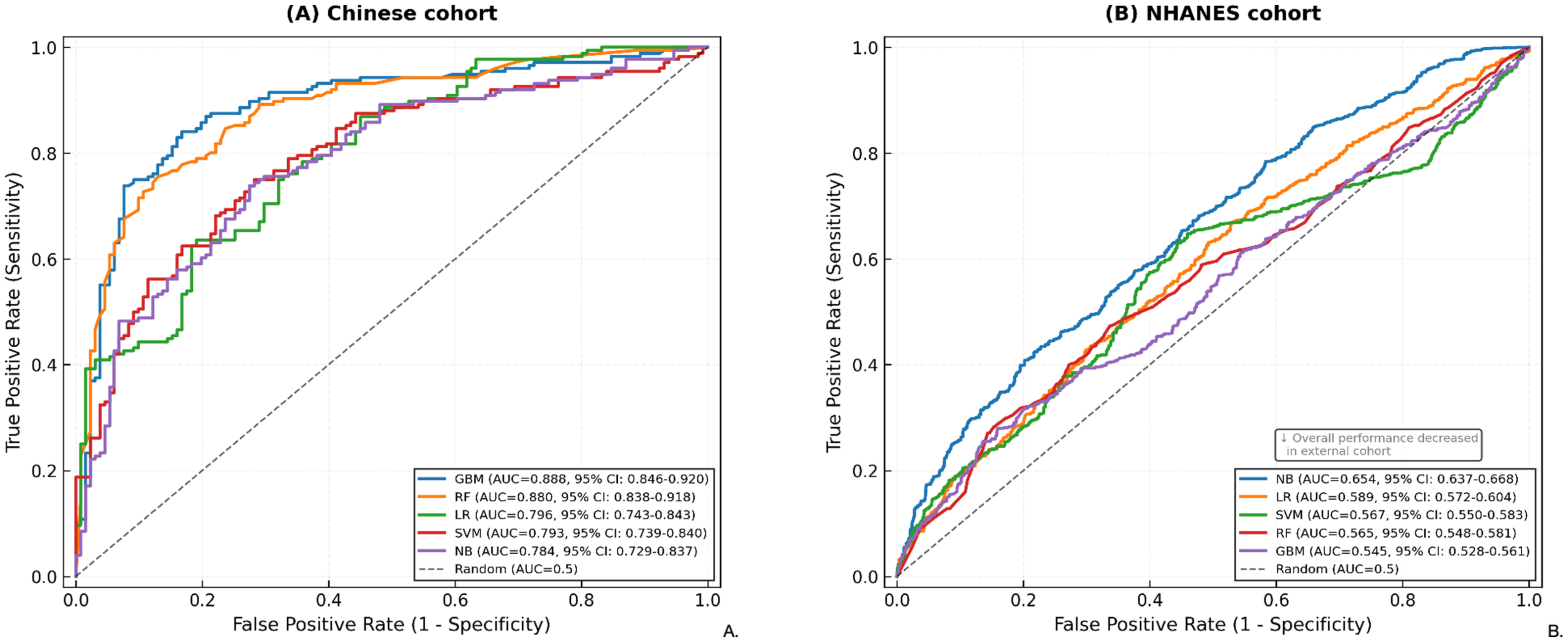
Comparison of machine learning model performance for predicting MASLD Risk. **(A)** Receiver operating characteristic (ROC) curves of five supervised machine learning models in the training cohort for predicting MASLD risk. **(B)** ROC curves of the same models in the external validation cohort (NHANES).

ROC curves for selected machine learning models are shown in Figure 5. In the Chinese training cohort (Figure 5A), models such as GBM and RF demonstrated strong discrimination with high AUC values. In contrast, ROC curves in the NHANES validation cohort (Figure 5B) showed reduced performance across all models, indicating limited generalizability. These findings highlight the challenges of cross-cohort prediction and emphasize the importance of external validation.

To compare the discriminative performance of different models, DeLong tests with bootstrap resampling were conducted (Supplementary Table S4). GBM achieved the highest AUC (0.8876, 95% CI, 0.8426–0.9211) and was considered the reference model. Compared with GBM, RF showed no statistically significant difference (*p* = 0.436), whereas other models, including XGBoost, MLP, KNN, logistic regression, SVM, Naïve Bayes, and decision tree, demonstrated significantly lower AUCs (all *p* < 0.05). These results indicate that GBM provides superior predictive performance in the Chinese cohort, although its advantage over RF was not statistically significant.

### Model calibration and clinical utility

The calibration performance of the machine learning model is shown in Figure 2A. The calibration curve demonstrated good agreement between predicted probabilities and observed outcomes, with most points closely aligned with the 45-degree reference line, indicating acceptable calibration of the model. DCA further evaluated the potential clinical utility of the model (Figure 2B). Across a range of threshold probabilities, the prediction model (“pred”) showed a higher net benefit compared with both the “treat-all” and “treat-none” strategies, suggesting that the model may provide added value for clinical decision-making.

### Model interpretation

**Figure 6:**
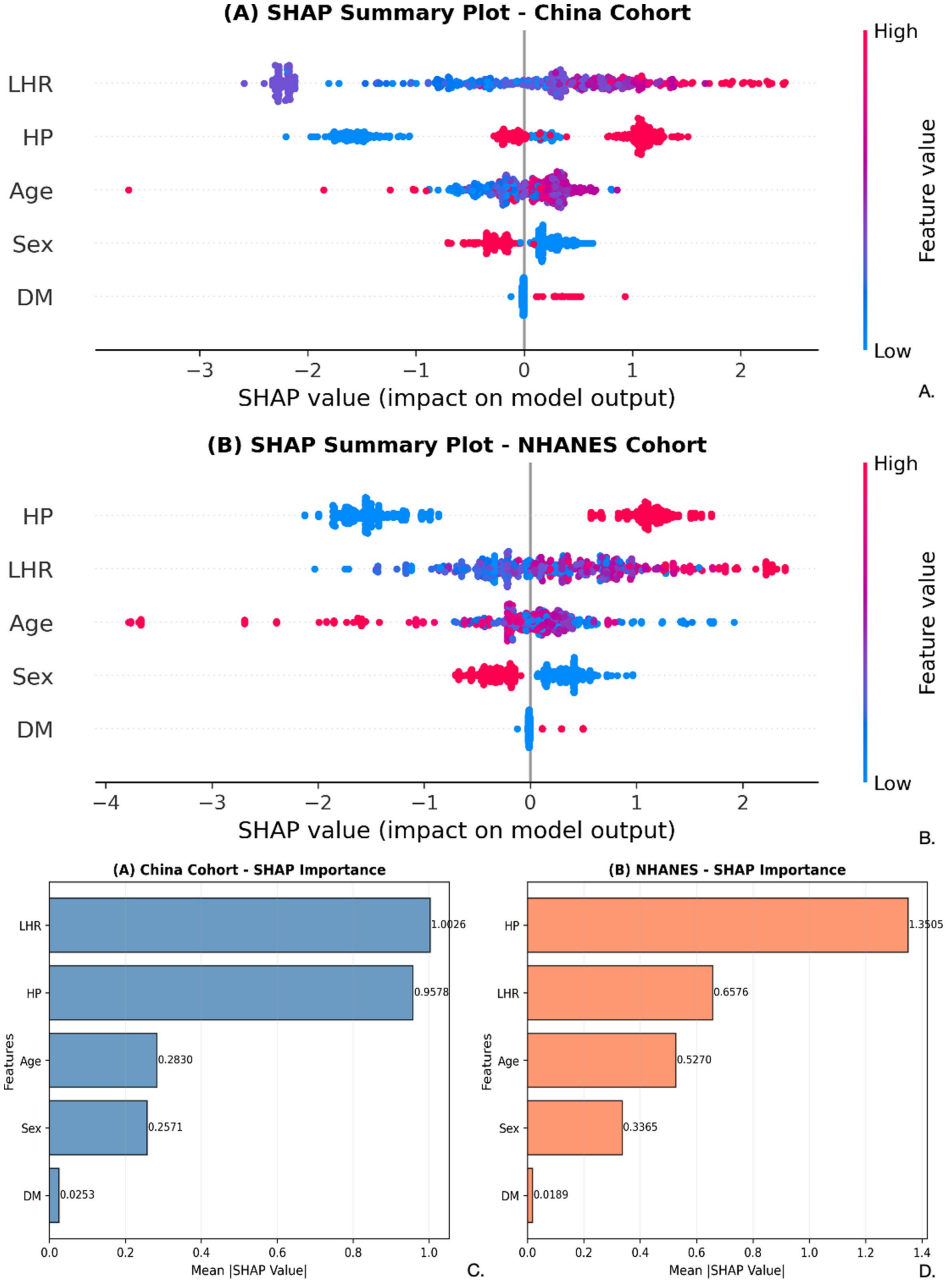
SHAP-based interpretation of machine learning models for MASLD prediction. **(A)** SHAP summary plot for the Chinese cohort. **(B)** SHAP summary plot for the NHANES cohort. **(C)** SHAP feature importance ranking in the Chinese cohort. **(D)** SHAP feature importance ranking in the NHANES cohort. Each dot in the SHAP summary plots represents an individual sample, with color indicating the feature value (red: high; blue: low) and position on the x-axis representing the SHAP value (impact on model output).

To further interpret the machine learning models, SHAP (Shapley Additive explanations) analysis was performed (Figure 6). In the Chinese cohort, the SHAP summary plot demonstrated that LHR was the most influential predictor of MASLD, followed by HP infection status, age, and sex. Higher LHR values and positive HP status were associated with an increased probability of MASLD.

In the NHANES cohort, SHAP analysis revealed a similar pattern, with HP infection and LHR remaining important contributors to model predictions, although the relative importance of features differed slightly compared with the Chinese cohort. These findings suggest that key predictors identified in the training cohort retained their relevance in the external population, supporting the robustness of the model.

The SHAP feature importance rankings (Figures 6C,D) further confirmed that metabolic and inflammatory indicators, particularly LHR, played a dominant role in MASLD risk prediction across cohorts.

To facilitate clinical application, a web-based MASLD risk prediction tool^1^ was developed based on the machine learning model (Supplementary Figure S2). The tool allows users to input key clinical variables, including age, sex, HP Infection status, DM, and LHR, to estimate the individual probability of MASLD. This application demonstrates the potential utility of the model for individualized risk assessment. However, given the cross-sectional nature of the data and differences between cohorts, the tool should be considered exploratory and requires further validation before clinical implementation. These findings highlight the potential of interpretable machine learning approaches in MASLD risk stratification.

## Discussion

In the present study, we observed an association between *Helicobacter pylori* (HP) infection and metabolic dysfunction-associated steatotic liver disease (MASLD) in both a Chinese hospital-based cohort and the NHANES population. Individuals with HP infection exhibited higher values of metabolic and inflammatory indices, including TyG, TyG/HDL, LHR, and NHR, indicating a shared metabolic-inflammatory profile (1, 4, 5).

FLI has known limitations compared with imaging. First, it can misclassify individuals with normal liver but high BMI or elevated TG (false positives), as well as those with steatosis but normal metabolic parameters (false negatives). Second, because FLI incorporates TG, BMI, GGT, and WC, it is partially determined by the same metabolic variables that we used as predictors in the Chinese cohort. This introduces non-differential misclassification and potential information leakage, both of which can attenuate true associations and limit comparability between cohorts. The use of FLI instead of ultrasound introduces misclassification bias. In general, such non-differential misclassification would bias the estimated OR toward the null, which may partly explain why the NHANES OR (1.22) is smaller than the Chinese OR (4.83 after full adjustment). However, the direction and magnitude of bias cannot be precisely quantified, and residual misclassification remains a limitation.

Multivariable logistic regression analyses indicated that HP infection remained significantly associated with MASLD after adjustment for demographic and selected clinical covariates in this cross-sectional setting. However, given the cross-sectional design and the possibility that several metabolic and inflammatory indices lie along the pathway between HP infection and MASLD, these results should be interpreted as exploratory and associative rather than causal (15). One hypothesized pathway involves HP infection and its potential relationship with insulin resistance (22), which may be explored in future longitudinal studies.

The magnitude of association in the Chinese cohort (OR = 4.83–6.48) was substantially higher than previously reported in other epidemiologic studies and meta-analyses (2, 3). Several factors may contribute to this discrepancy. First, participants were recruited from a hospital health examination center, which may introduce selection bias and enrich co-occurrence of HP infection and metabolic abnormalities (6, 8, 9). Therefore, the magnitude of the association observed in the Chinese cohort may reflect cohort-specific factors and should not be directly extrapolated to other populations. Second, household clustering or shared lifestyle factors may amplify observed associations. Third, residual confounding by unmeasured factors, such as diet, socioeconomic status, or genetic predisposition, cannot be excluded. Finally, differences in HP ascertainment (active urea breath test vs. serology) and MASLD definitions (ultrasound vs. FLI) may also contribute to cohort-specific effect sizes (17, 18, 20, 21). These considerations highlight that the Chinese cohort findings may not be generalizable to other populations.

Previous studies have reported inconsistent associations, highlighting heterogeneity across populations (13, 14). Specifically, some Asian cohorts reported a positive HP-MASLD association, while some Western cohorts did not observe a significant relationship (1, 4, 5). Evidence regarding HP eradication remains inconclusive. Reverse causality is also plausible, whereby metabolic abnormalities in MASLD patients may affect HP susceptibility or immune responses, influencing serological indicators. Thus, our findings should be interpreted in the context of existing evidence and highlight the need for future studies across diverse populations and interventional settings.

Differences in HP assessment (active urea breath test vs. serology) and MASLD definitions (ultrasound vs. FLI) further complicate cross-cohort comparisons (16, 17). In NHANES 1999–2000, imaging-based assessment was unavailable, and FLI was used as a surrogate, incorporating TG, BMI, GGT, and waist circumference, partially overlapping with model predictors, potentially contributing to cohort-specific signal capture and limited transportability (19, 20). Although variables directly derived from TG and WC were excluded in external validation, information leakage and misclassification cannot be fully ruled out. Because FLI is not equivalent to ultrasound, the external comparison between the Chinese cohort (ultrasound) and NHANES (FLI) should be viewed as an exploratory cross-cohort comparison rather than a true external validation. This analysis yielded lower AUC values (0.545–0.6536), indicating limited transportability and generalizability. Therefore, the predictive performance of machine learning models should be interpreted as exploratory and cohort-specific rather than immediately applicable to clinical practice (15). Furthermore, the NHANES 1999–2000 data reflect metabolic epidemiology from over two decades ago, a period with lower obesity prevalence and different diagnostic standards for steatotic liver disease. Consequently, the external validation results may not fully generalize to contemporary populations, and the modest AUCs could partly reflect these temporal shifts rather than purely model performance. Future validation using more recent cohorts with both HP testing and imaging-based MASLD assessment is needed.

Mediation analyses suggested that several metabolic and inflammatory indices, including TyG, TyG/HDL-C, LHR, NHR, and MHR, may contribute to the observed associations. Mediation results, derived from 1,000 bootstrap simulations and standardized indirect effects, should be interpreted as exploratory and hypothesis-generating rather than causal. However, due to the cross-sectional nature of the data, causal inference is not supported. The results should be considered hypothesis-generating and require validation in longitudinal or interventional studies. SHAP analysis identified LHR and HP infection as key contributors to model predictions. Importantly, SHAP values reflect contribution to model prediction, not mechanistic causality, and their importance may be cohort specific.

### Strengths and limitations

This study integrates a Chinese hospital-based cohort and an independent NHANES population, providing internal and external perspectives. Multiple machine learning algorithms were systematically compared, and SHAP analyses enhanced interpretability. Limitations include the cross-sectional design, which precludes causal inference; differing HP and MASLD assessments across cohorts; the lack of imaging in NHANES and the consequent reliance on FLI (a major limitation, as FLI is not interchangeable with ultrasound, which limits the generalisability of our external findings); potential multicollinearity among metabolic and inflammatory indices; modest external validation performance (NHANES AUC 0.545–0.6536), indicating limited transportability; residual confounding; selection bias in the Chinese cohort, meaning that the elevated OR (4.83–6.48) is likely cohort-specific and should not be generalised to the general population or to other ethnic groups; and the older temporal frame of NHANES 1999–2000, which may not reflect contemporary metabolic epidemiology or MASLD diagnostic frameworks (6, 8). Future studies should validate the machine learning models in cohorts with uniform, imaging-based MASLD assessment.

## Conclusion

HP infection is associated with MASLD within a shared metabolic-inflammatory profile. Findings are exploratory and hypothesis-generating. Machine learning models demonstrated limited predictive utility for risk stratification, with LHR and HP infection, along with TyG, LSR, and SIRI, identified as candidate predictors.

## Data Availability

The original contributions presented in the study are included in the article/Supplementary material, further inquiries can be directed to the corresponding author.

## Footnotes

1
https://masld-predictor-fvr5vp6jedjhvvv7sbwebv.streamlit.app
