# Supplementary material for: Association between Helicobacter pylori infection and MASLD: a cross-sectional study with interpretable machine learning for MASLD risk prediction
Source: Front Nutr. 2026 Jun 22;13:1730461. doi: 10.3389/fnut.2026.1730461 (PMC13333482; doi:10.3389/fnut.2026.1730461)
Supplement: Supplementary file 1 [file Table_1.DOCX]

Supplementary Table 1. Hyperparameters used for each machine learning model in the Chinese cohort

| Model | Hyperparameter | Value used | Note |
| --- | --- | --- | --- |
| GBM | n_estimators | 100 | Default; tuned via 5-fold CV |
|  | max_depth | 3 | Default |
|  | learning_rate | 0.1 | Default |
| RF | n_estimators | 100 | Default; tuned via 5-fold CV |
|  | max_depth | None | Default (trees expanded fully) |
|  | min_samples_split | 2 | Default |
| XGB | n_estimators | 100 | Default; tuned via 5-fold CV |
|  | max_depth | 6 | Default |
|  | learning_rate | 0.3 | Default |
| SVM | C | 1.0 | Default; tuned via 5-fold CV |
|  | kernel | rbf | Fixed |
|  | gamma | scale | Default |
| LR | C | 1.0 | Default; tuned via 5-fold CV |
|  | penalty | l2 | Default |
|  | max_iter | 1000 | Sufficient for convergence |
| MLP | hidden_layer_sizes | (50,) | Fixed |
|  | activation | relu | Default |
|  | alpha | 0.0001 | Default |
|  | max_iter | 1000 | Sufficient for convergence |
| KNN | n_neighbors | 5 | Default; tuned via 5-fold CV |
|  | weights | uniform | Default |
| NB | - | - | No hyperparameters |
| DT | max_depth | 5 | Fixed (to prevent overfitting) |
|  | min_samples_split | 2 | Default |
| GP | kernel | 1.0 * RBF(1.0) | Fixed |
| NN | hidden_layer_sizes | (100, 50) | Fixed |
|  | activation | relu | Default |
|  | alpha | 0.0001 | Default |
|  | max_iter | 1000 | Sufficient for convergence |
| Note: Hyperparameters were either set to default values (scikit-learn v1.3) or fixed as shown. Five-fold cross-validation within the training set (70% of Chinese cohort) was used to evaluate performance; grid search was not performed due to computational constraints and satisfactory baseline performance. Final models were retrained on the full training set using the listed parameters. | | | |

Supplementary File Table 2. Baseline characteristics according to MASLD (NHANES)

| Variables | Total (n = 4870) | Yes (n = 1989) | No (n = 2881) | Statistic | P |
| --- | --- | --- | --- | --- | --- |
|  |  |  |  |  |  |
| Age, M (Q₁, Q₃) | 49.00 (22.00, 66.00) | 57.00 (42.00, 68.00) | 36.00 (17.00, 63.00) | Z=-19.12 | <0.001 |
| Gender, n (%) |  |  |  | χ²=28.86 | <0.001 |
| Female | 2231 (45.81) | 1003 (50.43) | 1228 (42.62) |  |  |
| Male | 2639 (54.19) | 986 (49.57) | 1653 (57.38) |  |  |
| Race, n (%) |  |  |  | χ²=5.89 | 0.117 |
| Mexican American | 1357 (27.86) | 519 (26.09) | 838 (29.09) |  |  |
| Non-Hispanic Black | 971 (19.94) | 396 (19.91) | 575 (19.96) |  |  |
| Non-Hispanic White | 2111 (43.35) | 893 (44.90) | 1218 (42.28) |  |  |
| Other | 431 (8.85) | 181 (9.10) | 250 (8.68) |  |  |
| Education, n (%) |  |  |  | χ²=175.35 | <0.001 |
| Below high school | 1468 (30.14) | 808 (40.62) | 660 (22.91) |  |  |
| High School or above | 3402 (69.86) | 1181 (59.38) | 2221 (77.09) |  |  |
| Marital, n (%) |  |  |  | χ²=167.81 | <0.001 |
| No | 2638 (54.17) | 856 (43.04) | 1782 (61.85) |  |  |
| Yes | 2232 (45.83) | 1133 (56.96) | 1099 (38.15) |  |  |
| PIR, n (%) |  |  |  | χ²=3.81 | 0.051 |
| Not poor | 3881 (79.69) | 1612 (81.05) | 2269 (78.76) |  |  |
| Poor | 989 (20.31) | 377 (18.95) | 612 (21.24) |  |  |
| HP, n (%) |  |  |  | χ²=22.48 | <0.001 |
| No | 2802 (57.54) | 1064 (53.49) | 1738 (60.33) |  |  |
| Yes | 2068 (42.46) | 925 (46.51) | 1143 (39.67) |  |  |
| Obesity, n (%) |  |  |  | χ²=2315.22 | <0.001 |
| No | 3329 (68.36) | 592 (29.76) | 2737 (95.00) |  |  |
| Yes | 1541 (31.64) | 1397 (70.24) | 144 (5.00) |  |  |
| Diabetes, n (%) |  |  |  | χ²=49.51 | <0.001 |
| No | 4740 (97.33) | 1897 (95.37) | 2843 (98.68) |  |  |
| Yes | 130 (2.67) | 92 (4.63) | 38 (1.32) |  |  |
| Cholesterolemia, n (%) |  |  |  | χ²=46.92 | <0.001 |
| No | 4305 (88.40) | 1683 (84.62) | 2622 (91.01) |  |  |
| Yes | 565 (11.60) | 306 (15.38) | 259 (8.99) |  |  |
| TyG, M (Q₁, Q₃) | 8.01 (7.55, 8.51) | 8.48 (8.09, 8.94) | 7.73 (7.34, 8.12) | Z=-37.47 | <0.001 |
| TyG/HDL, M (Q₁, Q₃) | 6.49 (5.13, 7.95) | 7.66 (6.11, 9.07) | 5.85 (4.77, 7.07) | Z=-27.54 | <0.001 |
| SIRI, M (Q₁, Q₃) | 1.06 (0.72, 1.57) | 1.17 (0.82, 1.72) | 0.97 (0.66, 1.47) | Z=-10.15 | <0.001 |
| WLR, M (Q₁, Q₃) | 3.42 (2.87, 4.22) | 3.56 (2.96, 4.31) | 3.34 (2.78, 4.16) | Z=-6.11 | <0.001 |
| NLR, M (Q₁, Q₃) | 2.00 (1.50, 2.74) | 2.16 (1.61, 2.78) | 1.93 (1.44, 2.67) | Z=-6.69 | <0.001 |
| WNR, M (Q₁, Q₃) | 1.69 (1.53, 1.91) | 1.66 (1.52, 1.84) | 1.73 (1.55, 1.96) | Z=-7.26 | <0.001 |
| WHR, M (Q₁, Q₃) | 5.38 (4.10, 7.11) | 6.49 (4.92, 8.23) | 4.79 (3.67, 6.24) | Z=-23.71 | <0.001 |
| MHR, M (Q₁, Q₃) | 0.43 (0.32, 0.58) | 0.50 (0.37, 0.66) | 0.39 (0.29, 0.51) | Z=-18.98 | <0.001 |
| NHR, M (Q₁, Q₃) | 3.12 (2.27, 4.37) | 3.84 (2.82, 5.07) | 2.72 (1.98, 3.76) | Z=-22.95 | <0.001 |
| LHR, M (Q₁, Q₃) | 1.54 (1.14, 2.05) | 1.80 (1.32, 2.31) | 1.40 (1.05, 1.84) | Z=-17.90 | <0.001 |
| WMR, M (Q₁, Q₃) | 12.50 (10.43, 14.83) | 12.67 (10.71, 15.22) | 12.25 (10.20, 14.67) | Z=-4.50 | <0.001 |
| NMR, M (Q₁, Q₃) | 7.22 (5.67, 9.25) | 7.60 (6.00, 9.57) | 6.94 (5.50, 9.00) | Z=-7.64 | <0.001 |
| LSR, M (Q₁, Q₃) | 0.92 (0.74, 1.12) | 1.06 (0.88, 1.26) | 0.82 (0.68, 1.00) | Z=-28.06 | <0.001 |
| Z: Mann-Whitney test, χ²: Chi-square test | | | | | |
| M: Median, Q₁: 1st Quartile, Q₃: 3rd Quartile | | | | | |

Supplementary File Table 3 Subgroup analyses

| Variables | n (%) | No | Yes | OR (95%CI) | P | P for interaction |
| --- | --- | --- | --- | --- | --- | --- |
|  |  |  |  |  |  |  |
| DM |  |  |  |  |  | 0.502 |
| No | 977 (95.69) | 418 | 559 | 7.07 (5.32 ~ 9.39) | <.001 |  |
| Yes | 44 (4.31) | 9 | 35 | 3.87 (0.68 ~ 21.93) | 0.126 |  |
| Gender |  |  |  |  |  | 0.002 |
| Woman | 574 (56.22) | 191 | 383 | 4.32 (2.96 ~ 6.29) | <.001 |  |
| Man | 447 (43.78) | 236 | 211 | 10.85 (6.95 ~ 16.94) | <.001 |  |
| BMI median |  |  |  |  |  |  |
| ≤24.21 | 510 (49.95) | 216 | 294 | 6.01 (4.08 ~ 8.84) | <.001 | 0.209 |
| >24.21 | 511 (50.05) | 211 | 300 | 8.60 (5.73 ~ 12.89) | <.001 |  |
| Age median |  |  |  |  |  | 0.348 |
| ≤42 | 508 (49.76) | 250 | 258 | 7.79 (5.25 ~ 11.56) | <.001 |  |
| >42 | 513 (50.24) | 177 | 336 | 5.95 (3.99 ~ 8.88) | <.001 |  |

Supplementary File Table 4: The result of the Delong test

| Model | AUC | CI_lower | CI_upper | Diff_vs_Best | p_value | Significant |
| --- | --- | --- | --- | --- | --- | --- |
| GBM | 0.8875780707841776 | 0.8425533255907823 | 0.9210908429761129 | 0 | 1 | No |
| RF | 0.8802697779319917 | 0.8409511185264411 | 0.9175944294753925 | 0.007308292852185927 | 0.436 | No |
| XGB | 0.8636146773074255 | 0.8206845691726304 | 0.9059616667862523 | 0.023963393476752137 | 0.024 | Yes |
| MLP | 0.8393910478834143 | 0.792802127492542 | 0.8788622945860494 | 0.048187022900763266 | 0 | Yes |
| KNN | 0.7989243580846634 | 0.7469935884080341 | 0.8481155730755614 | 0.08865371269951416 | 0 | Yes |
| LR | 0.796321998612075 | 0.7394555178102562 | 0.8409460108500759 | 0.09125607217210263 | 0 | Yes |
| SVM | 0.7927220680083276 | 0.7439946414216317 | 0.841993945356636 | 0.09485600277585005 | 0 | Yes |
| NB | 0.7837873004857737 | 0.7320489919131494 | 0.8312777979586774 | 0.10379077029840389 | 0 | Yes |
| DT | 0.7561806037473976 | 0.7038895849334118 | 0.8052781293472021 | 0.13139746703678 | 0 | Yes |

Supplementary Table 5. Multiple imputation parameters for missing LHR in the NHANES cohort.

| Parameter | Value |
| --- | --- |
| Missing variable | LHR (lymphocyte/HDL ratio) |
| Number of missing values | 34 (0.7%) |
| Imputation method | Multiple Imputation by Chained Equations (MICE) |
| Software / function | Python sklearn.impute.IterativeImputer |
| Number of imputations | 5 |
| Maximum iterations per imputation | 10 |
| Predictor variables for imputation | Age, Sex, HP, DM, Cholesterolemia, TyG, MASLD |
| Imputation model for LHR | Linear regression |
| Pooling of results | Rubin’s rules |
| Sensitivity analysis | Complete-case analysis (excluding 34 cases) gave consistent results |


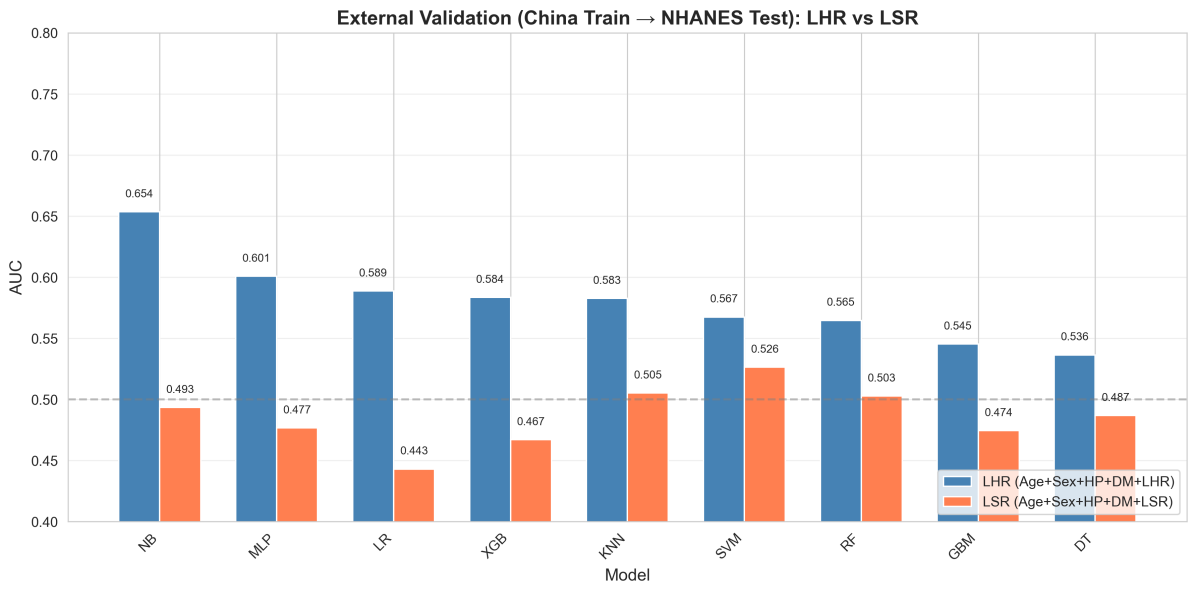


Figure S1. Exploratory cross‑cohort performance comparison between LHR‑based and LSR‑based models (Chinese training → NHANES).


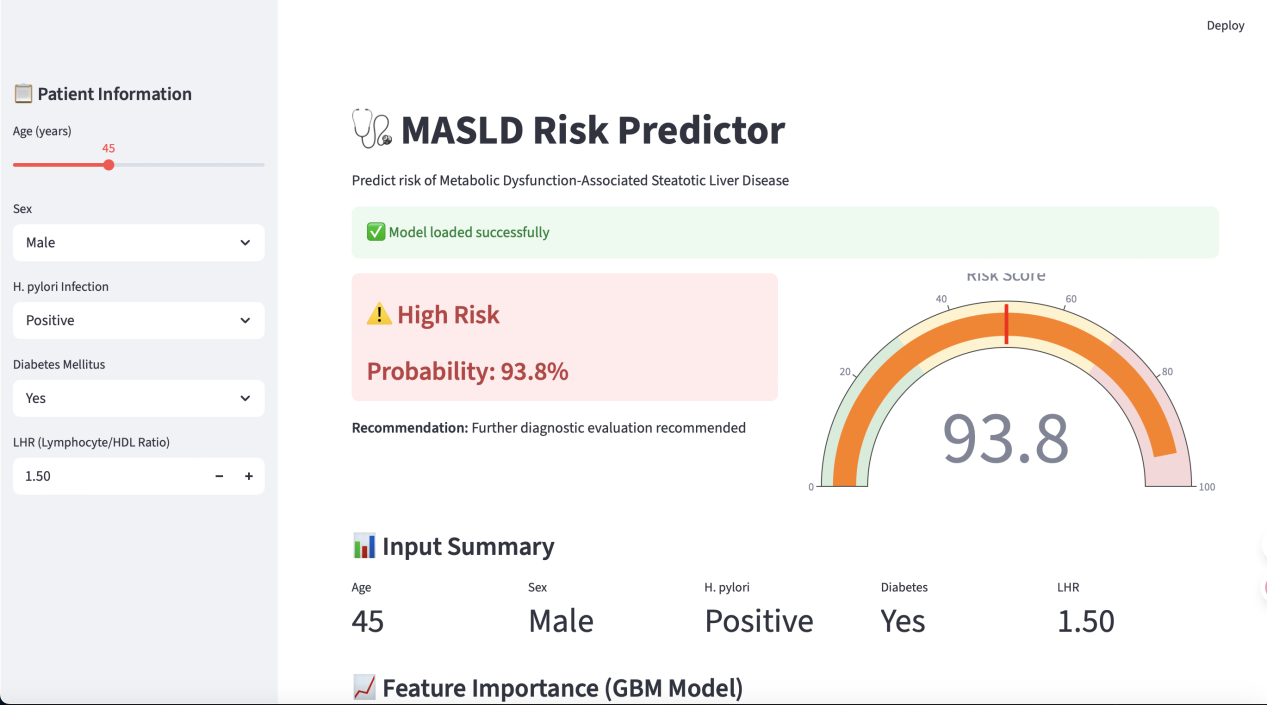


Figure S2. Web-based MASLD risk prediction tool based on the machine learning model. The interface allows users to input key clinical variables, including age, sex, Helicobacter pylori (HP) infection status, diabetes mellitus (DM), and LHR (lymphocyte-to-HDL ratio), to estimate the individual probability of MASLD.
